# The Mechanism of Action of Stigmasterol in Bone Formation in Osteoporosis

**DOI:** 10.3390/cimb48030337

**Published:** 2026-03-23

**Authors:** Cailian Lu, Hong Li, Zhengbo Liu, Sirui Lü, Junxing Liu

**Affiliations:** 1 Key Laboratory of Microecology-Immune Regulatory Network and Related Diseases, School of Basic Medicine, Jiamusi University, Jiamusi 154000, China; 2 The Cell Biology Laboratory, School of Basic Medical Sciences, Southern Medical University, Guangzhou 510515, China

**Keywords:** stigmasterol, osteoporosis, network pharmacology, JAK2/STAT3 signaling pathway

## Abstract

Osteoporosis (OP) is a metabolic bone disease characterized by reduced bone mass and impaired bone microarchitecture, significantly impacting patients’ quality of life. Stigmasterol (STG), a natural plant sterol, has been reported to possess multiple biological activities. However, its effects on OP bone formation and underlying molecular mechanisms remain unclear. The effects of STG on OP bone formation and potential mechanisms were investigated through in vivo and in vitro experiments combined with network pharmacology analysis. An OP model was established in ovariectomized (OVX) rats, and the bone-protective effects of STG were evaluated via micro-CT analysis and histological staining. In vitro experiments, MC3T3-E1 pre-osteoblasts were used to assess STG’s influence on osteogenic differentiation through Western blot analysis and ALP/ARS staining. Network pharmacology methods were used to predict potential targets and signaling pathways for STG in OP treatment, followed by mechanism validation. STG significantly improved bone microarchitecture in OVX rats, increased key osteogenic marker expression, and promoted MC3T3-E1 osteogenic differentiation in a dose-dependent manner. Network pharmacology analysis predicted 278 potential targets for STG in treating OP, with pathway enrichment analysis indicating significant involvement of the JAK/STAT pathway. Mechanistic studies revealed that STG promotes osteogenic differentiation by activating the JAK2/STAT3 signaling cascade. As an osteogenic promoter, STG effectively alleviates bone loss and enhances osteoblast differentiation by activating the JAK2/STAT3 signaling pathway.

## 1. Introduction

Osteoporosis (OP) is a chronic systemic skeletal disorder characterized by reduced bone mass, impaired bone microarchitecture, and diminished bone strength. Its pathogenesis involves endocrine factors, inflammatory and immune responses, and oxidative stress. Globally, this disease primarily affects the elderly population, particularly postmenopausal women [1,2,3]. Novel therapeutic agents for OP continue to emerge, with the primary objective of restoring equilibrium between bone resorption and formation. Selective estrogen receptor modulators (SERMs) and bisphosphonates are widely used in clinical practice, but their long-term use causes numerous adverse effects, such as esophagitis and osteonecrosis of the jaw [4,5]. Therefore, there is an urgent need to identify new targets and develop novel drugs to improve bone density and quality.

Stigmasterol (STG) is a plant sterol, belonging to the tetracyclic triterpenoid sterol group, and it is structurally similar to cholesterol. The human body cannot synthesize this sterol and must obtain it through diet, with common sources including vegetable oils (canola and soybean oils), grains, vegetables, unpasteurized milk, seeds, medicinal plants, nuts, and legumes [6]. Chemically, STG appears as a white crystalline powder with a density slightly higher than water [7]. It exhibits excellent solubility in organic solvents, dissolving readily in ethyl acetate, benzene, chloroform, and pyridine; is slightly soluble in acetone and ethanol; and is poorly soluble in water. STG exhibits chemical stability under standard temperature and pressure but may undergo saponification or complexation reactions under acidic or alkaline conditions, providing the chemical basis for its applications as a food emulsifier and drug carrier [8]. The medicinal effects of STG have been extensively reviewed in published literature [9,10]. In vitro and in vivo studies have demonstrated that STG possesses multiple pharmacological properties, including anticancer, anti-osteoarthritis, anti-inflammatory, antidiabetic, immunomodulatory, antiparasitic, antibacterial, antioxidant, and neuroprotective properties [11,12,13,14]. Current research indicates that STG significantly ameliorates osteoarthritis symptoms in collagen-induced arthritis (CIA) models [15]. Furthermore, STG effectively inhibits osteoclastogenesis and prevents bone loss by regulating the NF-κB and MAPK signaling pathways [16].

The JAK2/STAT3 signaling pathway has been established as a key component in bone formation and homeostasis maintenance [17]. This pathway belongs to the JAK/STAT family, within which JAK2 is widely expressed in various cells and tissues [18]. The STAT3 structure comprises multiple functional domains, including an N-terminal tetramerization domain, DNA-binding domain, SH2 domain, and C-terminal transcription activation domain [19]. STAT3 is expressed in diverse tissues such as bone and lung, and it plays a role in physiological and pathological processes, including early embryonic development; inflammatory responses; and cell proliferation, differentiation, and apoptosis [20]. JAK2 deficiency disrupts signaling between the growth hormone receptor (GHR) and its downstream mediator STAT, thereby impairing normal skeletal development [21]. STAT3 mediates anabolic signaling within osteoblasts and regulates the osteogenic process. Selective inactivation of STAT3 inhibits bone formation, resulting in reduced bone mass and increased susceptibility to traumatic fractures. Upon stimulation by cytokines, activated JAKs become phosphorylated, recruit STAT3 through SH2 domain binding, and catalyze the phosphorylation of specific tyrosine residues in the cytoplasmic tail of STAT3. Activated STAT3 dimerizes and translocates to the nucleus, where it binds to target gene promoter sequences to induce gene expression [21,22]. This enhances the transcription of osteogenesis-related genes such as RUNX2, OSX, ALP, and OCN, thereby promoting bone matrix mineralization [23]. Not only does this pathway respond to inflammatory signals, but its abnormal activation is also closely associated with skeletal diseases such as osteoarthritis and OP [24]. Therefore, precise regulation of JAK2/STAT3 signaling is crucial for maintaining normal bone metabolism and repair.

Despite significant progress has been made in STG’s inhibition of osteoclastogenesis [16], its regulatory role in bone formation remains poorly characterized. This study aims to evaluate STG’s bone-promoting effects through in vivo and in vitro experiments and to predict key signaling pathways for STG-mediated OP treatment via network pharmacology analysis, providing theoretical support for developing novel anti-osteoporosis drugs.

## 2. Materials and Methods

### 2.1. Principal Experimental Reagents and Instruments

After making a 10 mM stock solution with ethanol, STG (98% purity; MCE, Monmouth Junction, NJ, USA) was kept at −20 °C. Solarbio (Beijing, China) provided PEG300 and STG (95% purity; Sigma, St. Louis, MO, USA). Cellmax (Beijing, China) provided fetal bovine serum (FBS), while Gibco (Grand Island, NY, USA) supplied MEMα. Beyotime Biotechnology (Shanghai, China) provided the RIPA lysis buffer, PMSF, BCA protein content detection kit, SDS-PAGE gel preparation kit, crystal violet staining solution, BCIP/NBT alkaline phosphatase (ALP) color development kit, and hematoxylin and eosin (H&E) staining kit. We purchased Alizarin Red Ssolution (ARS) from Oricellbio (Guangzhou, China). Osteogenic induction medium was sourced from Xrbio (Hangzhou, China). The reverse transcription kit was purchased from Vazyme Biotechnology (Nanjing, China).

The primary antibodies utilized in this study included β-actin (ZSGB-BIO, #TA-09, Beijing, China), Osteocalcin (ABclonal, #A20800, Wuhan, China), RUNX2 (ABclonal, #A11753), STAT3 (ABclonal, #A1192), Phospho-STAT3 (Tyr705) (ABclonal, #AP0705), JAK2 (Cell Signaling Technology, #3230), and Phospho-JAK2 (Tyr1007/1008) (Cell Signaling Technology, #3776, Danvers, MA, USA). The Cell Counting Kit-8 (CCK-8) reagent was purchased from MeilunBio (Dalian, China).

### 2.2. Identification and Screening of Potential STG Targets

Compounds obtained from the PubChem database (https://pubchem.ncbi.nlm.nih.gov, accessed on 22 March 2025) were input as SMILES into Swiss Target Prediction (http://www.swisstargetprediction.ch, accessed on 22 March 2025) to forecast their potential protein targets, utilizing the COREMINE database (https://coremine.com, accessed on 22 March 2025). All predicted targets with a “probability value of 0” were carefully eliminated from subsequent analyses to ensure the dependability of the candidate targets.

### 2.3. Acquisition of OP Target Genes

The keyword “Osteoporosis” was entered into the GeneCards (https://www.genecards.org/, accessed on 22 March 2025) and OMIM databases to search for target genes.

### 2.4. Construction of the Common Target and PPI Network of STG and OP

A Venn diagram was generated using the Weishengxin Data Analysis Platform (http://www.bioinformatics.com.cn/, a free online platform for bioinformatics analysis and visualization, accessed on 22 March 2025) to illustrate the overlapping targets between STG and OP. The common targets obtained from the intersection were subsequently imported into the STRING database (version 11.0, http://string-db.org, accessed on 22 March 2025) to construct a protein–protein interaction (PPI) network. The search was restricted to *Homo sapiens*, with a threshold of a minimum interaction score of 0.7 (high confidence). The resultant PPI network was then imported into Cytoscape software version 3.9.0 for further visualization and topological analysis. A key topological index, the node degree, was calculated. To identify the important targets in the network, their visual attributes were adjusted, such as their size and color, according to the degree value of each node.

### 2.5. GO and KEGG Pathway Enrichment Analyses

The Gene Ontology (GO) database (http://www.geneontology.org, accessed on 22 March 2025) comprises experimental results from different species, thus facilitating the exploration of terms related to cellular components (CCs), molecular functions (MFs), and biological processes (BPs) in a huge gene dataset. GO enrichment analysis can reveal the locations of many genes in cells, their functional roles, and the biological activities involved. Kyoto Encyclopedia of Genes and Genomics (KEGG, https://www.genome.jp/kegg/, accessed on 22 March 2025) provides the latest functional annotations and serves as a resource for the systematic study of gene functions. The combination of GO and KEGG enrichment analyses can aid in the identification of signaling networks between drugs and diseases, thereby providing a more comprehensive understanding of the function of genes. The intersection targets were imported into the Weishengxin platform for GO and KEGG enrichment analyses, and the obtained data were visualized using GO and KEGG bubble charts.

### 2.6. Molecular Docking

The 3D structure of the target protein (also called a receptor) was obtained from the UniProt database (https://www.uniprot.org/, accessed on 23 March 2025). The 3D structure of the ligand STG was downloaded from the PubChem database (https://pubchem.ncbi.nlm.nih.gov, accessed on 23 March 2025). PyMOL software version 2.6.0 was used to remove unwanted water molecules and other small molecules from the receptor structure. Next, AutoDockTools 1.5.7 was used to prepare the receptor and ligand for molecular docking. This included setting the “root” of the ligand and which bonds can be rotated, adding polar hydrogen atoms to it, and calculating the Gasteiger charge. The real molecular docking simulation was completed using AutoDock Vina software v1.2.7. Finally, PyMOL software was used to open, view, and carefully examine the possible docking combinations.

### 2.7. Cell Culture and Treatment

Haixing Biotechnology Co., Ltd. (Suzhou, China) provided the MC3T3-E1 mouse pre-osteoblast cell line. MEMα medium supplemented with 10% fetal bovine serum (FBS) was used to culture the cells, which were kept at 37 °C in a humidified environment with 5% CO_2_. The cells were passaged or cryopreserved when reaching approximately 90% confluence for subsequent experimental use.

### 2.8. Induction of the OP Model and Drug Treatment in Animals

An OP model was established in animals with bilateral ovariectomy. This investigation employed forty 8-week-old SPF female SD rats, each weighing 200 ± 20 g, obtained from Changchun Yisi Experimental Animal Technology Co., Ltd. (License No.: SCXK (Ji) 2023-0002, Changchun, China). After one week of acclimatization, the rats were maintained under regulated conditions (23 °C, 12-h light/dark cycle) at the Jiamusi University Laboratory Animal Center (License No.: SYXK (Hei) 2021-018). The Jiamusi University Animal Experiment Ethics Committee approved all animal experiments (Approval No.: JDJCYXY20240052; Date of Approval: 10 October 2024).

Ovariectomy (OVX) was employed to simulate OP. In short, the rats were given an intraperitoneal injection of 50 mg/kg of 1% pentobarbital sodium to induce anesthesia. Following anesthesia, the peritoneal cavity was exposed by shaving the dorsal hair and making a skin and muscle incision at the costal angle. The uterine horns were identified, and the adipose tissue surrounding the ovaries was carefully exposed. The ovaries were located, and they had a cauliflower-like shape. Before the ovaries were removed, ligation was performed at the uterine horns and the upper and lower parts of the fallopian tubes. After the ovaries were taken out, the muscles and skin tissues were sewn in turn with stitches. The adipose tissue was repositioned, and the incision was closed. Penicillin was administered daily for seven consecutive days to prevent postoperative infection. The wounds were regularly cleaned, recovery was carefully monitored after the operation, and any symptoms or unusual behaviors were recorded. The rats were subsequently allocated into five groups (*n* = 8 per group): (1) sham-operated group (Sham), (2) OVX model group (OVX), (3) OVX + low-dose STG (10 mg/kg), (4) OVX + medium-dose STG (20 mg/kg), and (5) OVX + high-dose STG (40 mg/kg). Given the poor solubility of STG in water and conventional aqueous solvents (solubility in water < 0.1 mg/mL), it was difficult to prepare a clear solution, so the drug was administered in the form of a suspension. Precisely weighed STG powder was added to a solution containing 0.5% Tween-80, 40% PEG300, and 60% normal saline. The mixture was magnetically stirred at room temperature for 30 min, followed by ice-bath ultrasonic dispersion for 10 min. No significant particle aggregation was observed in the suspension, resulting in homogeneous suspensions with concentrations of 2, 4, and 8 mg/mL. The addition of Tween-80 effectively improved the dispersion stability of STG, preventing sedimentation during storage and administration, as well as ensuring the accuracy of the intragastric dosage (5 mL/kg body weight). The sham and OVX groups were administered an isovolumetric solution containing 0.5% Tween-80, 40% PEG300 and 60% normal saline as a carrier control. The duration of this treatment was twelve weeks. After 12 weeks of treatment, the animals were anesthetized with an overdose of 1% pentobarbital sodium and euthanized. Blood samples were collected from the abdominal aorta, allowed to clot at room temperature, and centrifuged at 1500 rpm for 10 min to separate the serum. Both femurs were rapidly dissected and stripped of soft tissue. One femur was fixed in buffered formalin for histopathological examination, while the contralateral femur was snap-frozen in liquid nitrogen and stored at −80 °C for subsequent biochemical analysis.

### 2.9. Cell Viability Assay (CCK-8)

MC3T3-E1 cells were planted in 96-well plates, with 5.0 × 10^3^ cells in each well, and left overnight so that they could stick well. Subsequently, the medium was replaced with drug-containing medium, and different concentration gradients of STG (0, 2.5, 5, 10, 15, 20, and 40 μM) were set. After 24 h of treatment, 10 μL of CCK-8 solution was added to each well. To determine how many cells were alive, the plate was placed in an environment at 37 °C for one hour, and then the optical density color of each well was measured at 450 nm with a machine.

### 2.10. Osteogenic Differentiation and Mineralization Assays

We planted MC3T3-E1 cells in 6-well plates, with 4.0 × 10^4^ cells in each well. After 24 h of growth, we replaced the original culture solution with osteogenic induction medium, which contained different concentrations of STG (0, 2.5, 5, 10 µM). We changed the culture medium every two or three days. To investigate alkaline phosphatase (ALP) activity, we let the cells grow for 14 days, then fixed them with 4% paraformaldehyde, and finally dyed them using a BCIP/NBT alkaline phosphatase chromogenic kit, according to the manufacturer’s instructions. To observe the mineralization of the matrix, we let the cells grow for 21 days, fixed them with 4% paraformaldehyde, and then dyed them with 1% Alizarin Red Solution (pH 4.2). After dyeing, we carefully washed them three times with distilled water to clean the areas that should not be dyed. Finally, we examined the stained cells were with an optical microscope and photographed them.

### 2.11. Western Blot

RIPA buffer with 1% PMSF was used to crush the cells on ice. The obtained liquid was first ultrasonically treated and then centrifuged at a speed of 12,000× *g* at 4 °C for 30 min. BCA detection kit was used to determine the protein concentration in the supernatant. After separating the same quantity of protein from each sample using SDS-PAGE, it was transferred to a PVDF membrane. It was sealed in 5% skim milk for an hour at room temperature to prevent the film from sticking. Next, it was incubated overnight at 4 °C with specific primary antibodies. After three washes, the membrane was incubated for an hour at room temperature with secondary antibodies labeled with HRP. The ECL substrate and chemiluminescence imaging method showed protein bands. Finally, ImageJ software version 1.54 was used to measure the intensity of these bands.

### 2.12. Micro-CT

After 12 weeks of drug treatment, we euthanized the rats using an intraperitoneal injection of pentobarbital sodium. Then, we carefully removed the femur and kept them in 4% paraformaldehyde solution for 48 h. We scanned the metaphyseal end of the distal femur of each sample with high precision using Imaging100 microcomputer tomography equipment (Raycision Medical Technology Co., Ltd., Hefei, China), with a voltage of 45 kV, a current of 435 mA, and a stereoscopic pixel size of 15 microns. To analyze the three-dimensional microstructure characteristics, including trabecular number, trabecular thickness, bone mineral density, and bone volume fraction, we used the software recommended by the equipment manufacturer.

### 2.13. Bone Histomorphometry and Immunohistochemistry

To determine whether STG can aid in the healing of OP rat femurs, a histological examination was conducted. First, the rat femoral samples were scanned using micro-CT and then soaked in 10% EDTA solution for three weeks to remove minerals. After complete decalcification, the tissue was washed with xylene, then slowly dehydrated with ethanol, wrapped in paraffin, and finally cut into 5-micron-thick slices with a microtome. Hematoxylin and eosin (H&E) staining was then applied to these slices, and immunohistochemistry (IHC) analysis was performed to look for certain markers of bone formation. Histopathological alterations were observed and visualized using a light microscope. The expression levels of osteoblast-related proteins in IHC-stained sections were assessed semi-quantitatively by examining staining intensity with ImageJ software.

### 2.14. Immunofluorescence

The tissue sections were dewaxed and rehydrated. Antigen retrieval was performed using solutions such as EDTA, followed by permeabilization with PBS containing 0.3% Triton X-100 and blocking with PBS containing 5% BSA. The primary antibody was diluted to ensure complete contact with the tissue sections and incubated overnight at 4 °C to facilitate specific binding to the target antigen. Following incubation, the sections were washed and incubated at room temperature with a fluorescently labeled secondary antibody for 1 h. Subsequent washes removed unbound antibodies and impurities. Finally, cell nuclei were stained with DAPI staining solution and observed under a fluorescence microscope.

### 2.15. EdU Staining

2 × 10^4^ cells were placed in each group of six-well plates. When these cells were 60% to 80% full, they were treated with different concentrations of STG. After 24 h in the dark, color changes were observed. The fluorescence intensity was measured with a fluorescence microscope, and photos were taken. The following formula was used to calculate the percentage of EdU-positive cells: (number of EdU positive cells/number of cells stained by DAPI) times 100%.

### 2.16. Statistical Analysis

All experiments were conducted independently at least three times. The normality of the data was assessed using the Shapiro–Wilk test. For data exhibiting a normal distribution, comparisons between two groups were performed using *t*-tests in SPSS 29 software, while a one-way analysis of variance was employed for comparisons among multiple groups. LSD post hoc tests were applied to data with homogeneous variance, and Dunnett T3 tests were utilized for data with unequal variance. Non-parametric tests were conducted for data that did not follow a normal distribution. A significance level of *p* < 0.05 was considered indicative of a significant difference in the results. Data are expressed as mean ± standard deviation (SD) using GraphPad Prism 8.0.1, with *n* = 3 samples per group.

## 3. Results

### 3.1. STG Alleviates Bone Loss and Ameliorates OP

#### 3.1.1. Validation of the Osteoporotic Animal Model

**Figure 1:**
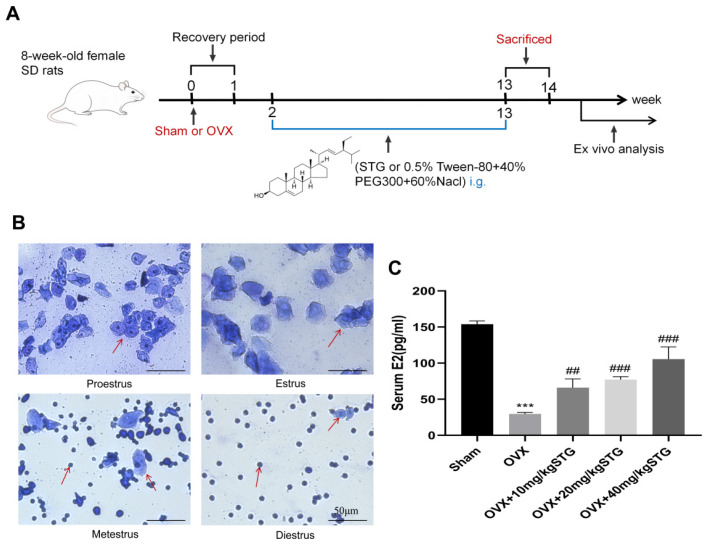
Validation of the osteoporotic animal model. (**A**) Chemical structure of STG and timeline of animal experiments. (**B**) Estrous cycle. (**C**) ELISA detection of serum estradiol concentration. Data are shown as the mean ± SD of three independent experiments. Compared with the sham group, *** *p* < 0.001; compared with the OVX group, ^##^ *p* < 0.01,^###^ *p* < 0.001. The red arrow points to: Proestrus: nucleated epithelial cells; Estrus: cornified (anucleated) epithelial cells; Metestrus: leukocytes, nucleated epithelial cells; Diestrus: leukocytes.

We employed vaginal cytology to monitor the estrous cycle in female rats, thereby confirming the successful establishment of the OVX-OP model. This cycle often lasts for four to five days and consists of four separate phases: (1) the proestrus phase: this is characterized by the presence of round or oval epithelial cells with nuclei, occasionally accompanied by a few keratinocytes and leukocytes; (2) the estrus phase: during this phase, the majority of cells are anucleate keratinocytes, often appearing in clusters; (3) the metestrus phase: this is marked by a significant influx of leukocytes intermixed with some nucleated epithelial cells and keratinocytes; (4) the diestrus phase: in this phase, the field of vision is predominantly filled with dense leukocytes, along with a small amount of viscous fluid (Figure 1B).

The results indicated that the estrous cycles of the rats in the sham operation group were consistent and relatively stable, whereas those in the OVX model establishment group remained in the proestrus and diestrus phases continuously. This observation suggested a disorder in their estrous cycles, leading to the initial conclusion that the OP model had been successfully established. Following the final administration, serum samples were collected from the rats in each group. The concentration of estradiol (E2) in the serum was determined using ELISA. E2, the primary estrogen in the body, plays a crucial protective role in maintaining bone mass. The findings revealed a significant decrease in serum E2 concentrations in the OVX group, and those in the OVX + 10 mg/kg STG, OVX + 20 mg/kg STG, and OVX + 40 mg/kg STG groups were significantly higher than those in the OVX group (Figure 1C).

#### 3.1.2. Effects of STG on Bone Structure and Morphology in OP Model Rats

**Figure 2:**
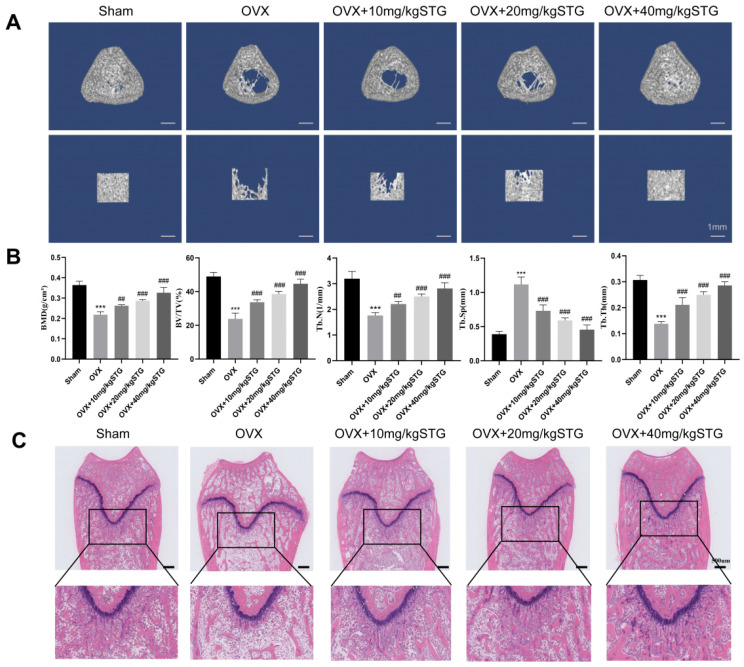
Effects of STG on bone structure and morphology in OP model rats. (**A**) Micro-CT image. (**B**) Quantitative analysis. (**C**) H&E staining. Data are shown as the mean ± SD of three independent experiments. Compared with the sham group, *** *p* < 0.001; compared with the OVX group, ^##^ *p* < 0.01,^###^ *p* < 0.001.

We assessed the impact of STG on the bone tissue structure in the OP model using micro-CT (Figure 2A,B). The analysis revealed that, compared to the sham group, the bone mineral density (BMD) in the ovariectomized (OVX) group decreased by 40%, the bone volume-to-total volume ratio (BV/TV) decreased by 51%, trabecular number (Tb.N) decreased by 44.8%, and trabecular separation (Tb.Sp) increased by 187%. These changes were statistically significant, confirming the successful establishment of the OP model. In comparison to the OVX group, the BMD, BV/TV, trabecular thickness (Tb.Th), and Tb.N in the OVX + 10 mg/kg STG, OVX + 20 mg/kg STG, and OVX + 40 mg/kg STG groups all increased, while Tb.Sp decreased. This indicates that treatment with STG can mitigate bone loss induced by OVX in a dose-dependent manner.

H&E staining revealed a significant decrease in trabecular bone and a more disorganized arrangement in the OVX group than in the sham group. In comparison to the OVX group, the trabecular bone in the OVX + 10 mg/kg, OVX + 20 mg/kg STG, and OVX + 40 mg/kg STG groups exhibited increased density and improved organization (Figure 2C).

#### 3.1.3. The Effect of STG on OCN and RUNX2 Expression in Bone Tissue of OP Model Rats

**Figure 3:**
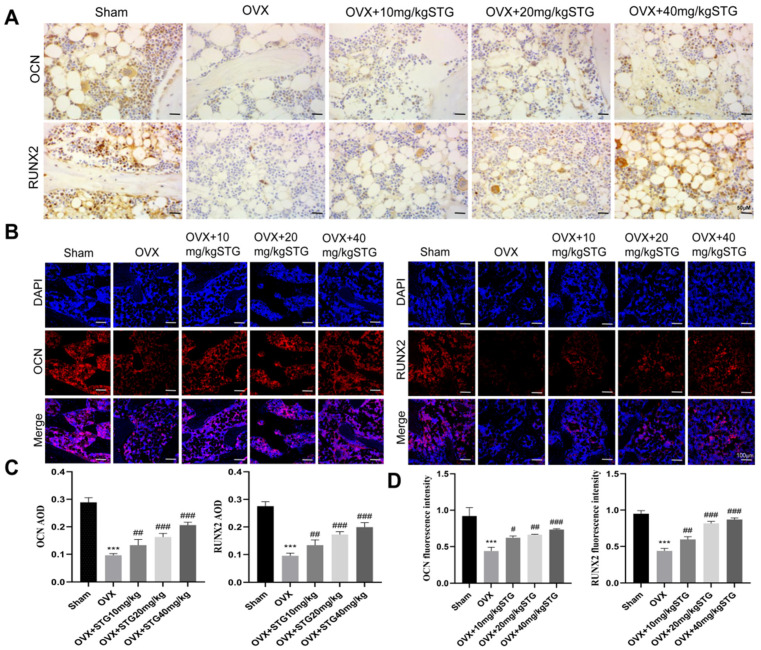
The effect of STG on OCN and RUNX2 expression in bone tissue of OP model rats. (**A**) IHC images of RUNX2 and OCN. (**B**) IF images of RUNX2 and OCN. (**C**) IHC and IF quantitative analyses of RUNX2 and OCN. Data are shown as the mean ± SD of three independent experiments. Compared with the sham group, *** *p* < 0.001; compared with the OVX group, ^#^ *p* < 0.05,^##^ *p* < 0.01,^###^ *p* < 0.001.

IHC and IF analyses indicated that (Figure 3), in comparison to the sham group, the specific staining of RUNX2 and OCN in the OVX group was markedly reduced. Conversely, the specific staining in the OVX + 10 mg/kg STG, OVX + 20 mg/kg STG, and OVX + 40 mg/kg STG groups was significantly enhanced relative to that in the OVX group. Collectively, these results suggest that STG administration can mitigate bone degradation resulting from reduced estrogen levels following oophorectomy and stimulate the synthesis of key osteogenic proteins.

### 3.2. Effects of STG on Cellular Activity

**Figure 4:**
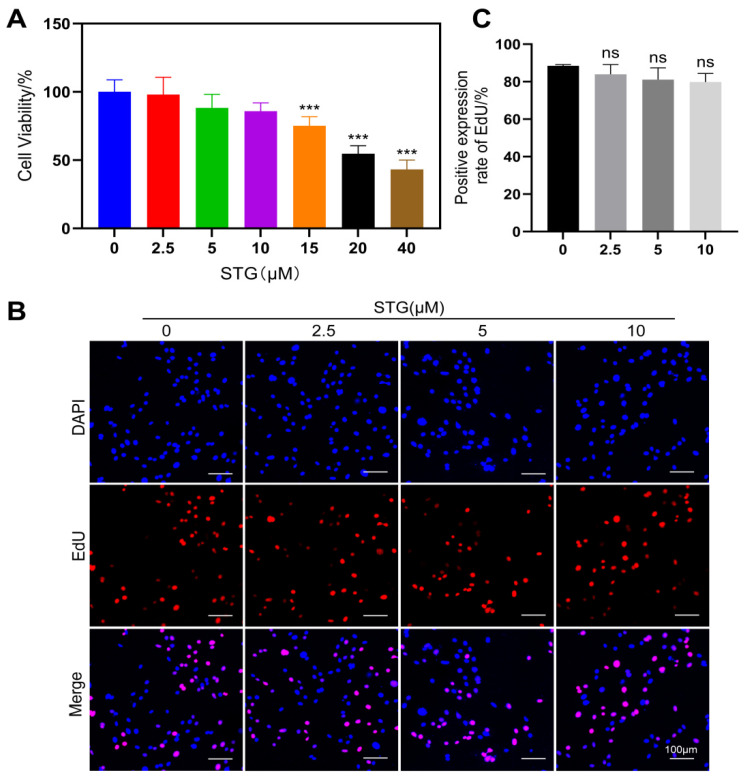
Effects of STG on the viability and proliferation of MC3T3-E1 cells. (**A**) Effects of different concentrations of STG on MC3T3-E1 cell viability detected using a CCK-8 assay. (**B**) Effects of different concentrations of STG on MC3T3-E1 cell proliferation detected using EdU staining. (**C**) Statistical analysis of EDU results. Data are shown as the mean ± SD of three independent experiments. “ns” indicates no statistical significance. Significance levels are denoted as *** *p* < 0.001.

To assess the impact of STG on the proliferation of MC3T3-E1 cells, we initially employed a CCK-8 assay to measure the changes in cell proliferation following treatment with varying concentrations of STG for 24 h. The results are presented in Figure 4A. STG concentrations ranging from 2.5 to 10 μM did not enhance cell proliferation or exhibit notable cytotoxic effects, whereas those ranging from 15 to 40 μM significantly reduced cellular activity. Additionally, the EdU assay results indicated no significant difference in cell proliferation between the STG-treated group and the control group (Figure 4B,C).

### 3.3. STG Promotes Osteogenic Differentiation of MC3T3-E1 Cells

**Figure 5:**
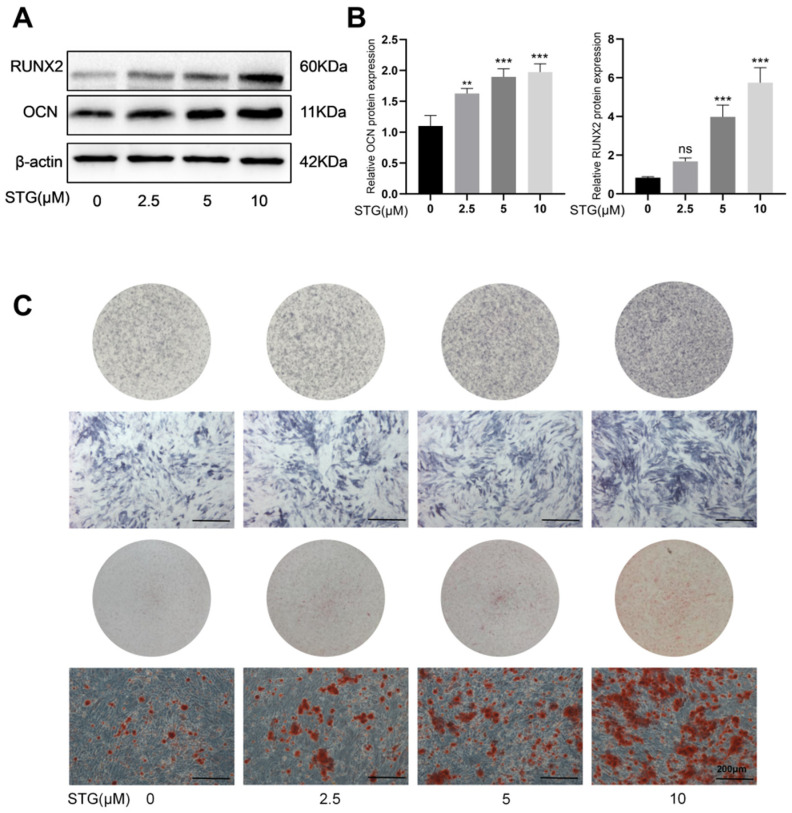
Effect of STG on osteoblast differentiation. (**A**) Effects of STG on the expression of osteogenesis-related proteins in cells. (**B**) Statistical analysis of OCN and RUNX2 proteins. (**C**) ALP and ARS staining showing the effects of different concentrations of STG on osteoblast differentiation. Data are shown as the mean ± SD of three independent experiments. “ns” indicates no statistical significance. Significance levels are denoted as ** *p* < 0.01, *** *p* < 0.001.

To observe the effects of STG on osteogenic differentiation, we treated MC3T3-E1 cells with different concentrations of STG under the condition of osteogenic induction. Western blot analysis revealed that, compared with the control group, 2.5–10 μM STG significantly increased the protein expression level of the osteogenic marker OCN, while the osteogenic marker RUNX2 showed a more pronounced expression at 5–10 μM (Figure 5A,B). Furthermore, ALP activity assessed on day 14 and matrix mineralization evaluated via ARS staining on day 21 were both markedly enhanced in the STG-treated groups compared to the control group (Figure 5C). These results indicate that STG can dose-dependently promote the osteogenic differentiation of MC3T3-E1 cells. However, its specific molecular targets and mechanisms of action remain unclear and require further investigation.

### 3.4. Network Pharmacology Analysis of STG and OP

#### 3.4.1. Identification of STG and OP-Related Targets

**Figure 6:**
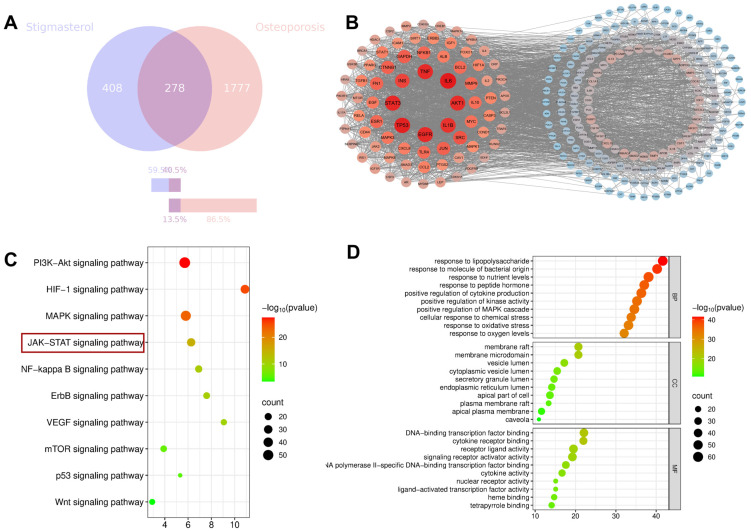
Network pharmacology workflow. (**A**) Venn diagram of STG and OP. (**B**) Protein–protein interaction network of common targets of STG and OP. (**C**) KEGG pathway enrichment analysis. (**D**) GO functional enrichment analysis.

Potential protein targets of STG were retrieved by integrating data from the COREMINE, TCMSP, and Swiss Target Prediction databases. After removing duplicate items, 686 different STG candidate targets were identified. At the same time, genes related to OP were obtained from the specialized databases of GeneCards and OMIM. After this, 2055 non-repetitive targets were identified. The common targets were obtained by intersecting the 686 STG targets with the 2055 OP-related targets. Visualization in a Venn diagram revealed 278 overlapping targets, which were identified as possible key targets for STG in OP treatment (Figure 6A).

#### 3.4.2. PPI Network

The 278 cross-target nodes in the figure were input into the STRING database (version 11.0), with the minimum interaction score threshold set to >0.7. Cytoscape was used to draw a network diagram of protein interactions, as shown in Figure 6B. There are 269 nodes and 3132 connecting lines in this network.

#### 3.4.3. GO and KEGG Pathway Enrichment Analysis

To further investigate the biological role of STG in the treatment of OP, we conducted Gene Ontology (GO) and Kyoto Encyclopedia of Genes and Genomes (KEGG) enrichment analyses of the target genes using the Weishengxin platform. As illustrated in Figure 6D, the GO biological process (GO-BP) analysis suggests that STG may be associated with several biological processes, including the positive regulation of kinase activity, the positive regulation of the MAPK cascade, the enzyme-linked receptor protein signaling pathway, the positive regulation of protein phosphorylation, the positive regulation of cell migration, and the response to oxidative stress. The GO cellular component (GO-CC) analysis indicates that the cellular components involved in STG’s action against OP are primarily located in membrane structures, vesicles, and the endoplasmic reticulum. The GO molecular function (GO-MF) analysis suggests that STG may be related to molecular functions such as protein kinase activity, growth factor binding, and cytokine receptor binding. Consequently, the genes regulated by STG are likely to play a significant role in the secretion of relevant cytokines and the regulation of protein activity. Figure 6C presents the pathways associated with STG in the treatment of OP, as identified through the KEGG enrichment analysis. These pathways include the PI3K-Akt, HIF-1A, MAPK, JAK-STAT, NF-κB, ErbB, and Wnt signaling pathways.

#### 3.4.4. Screening of Key Targets

**Figure 7:**
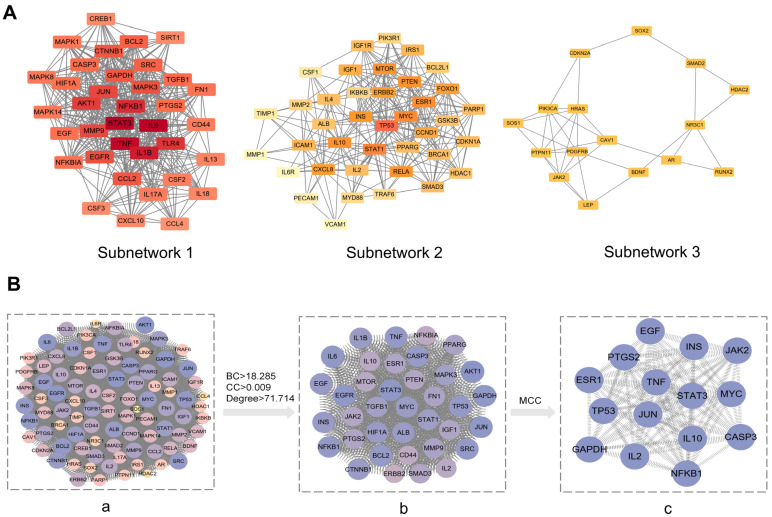
Screening of key targets. (**A**) Top 3 subnetworks. (**B**) (**a**): PPI network diagram of three sub-networks. (**b**): Topological analysis results of betweenness centrality, closeness centrality, and degree centrality. (**c**): MCC analysis results.

Cluster analysis was performed using the MCODE plugin in Cytoscape 3.9.0 software, with the top three subnetworks selected (Figure 7A), yielding a total of 91 key targets. Details are provided in Table 1. The betweenness centrality, closeness centrality, and degree centrality of each network node were analyzed using the topological features of Cytoscape 3.9.0. The results of this topological analysis revealed that the target proteins with higher degree values included EGF, TP53, AKT1, and STAT3. Subsequently, the CytoHubba plugin was employed to screen the top 15 network nodes using the MCC method, which were classified as the hub genes associated with STG in the treatment of OP. These hub genes included STAT3, JUN, TP53, ESR1, EGF, TNF, MYC, IL2, and JAK2 (Figure 7B).

### 3.5. STG Promotes Osteogenic Differentiation via the JAK2/STAT3 Signaling Pathway

**Figure 8:**
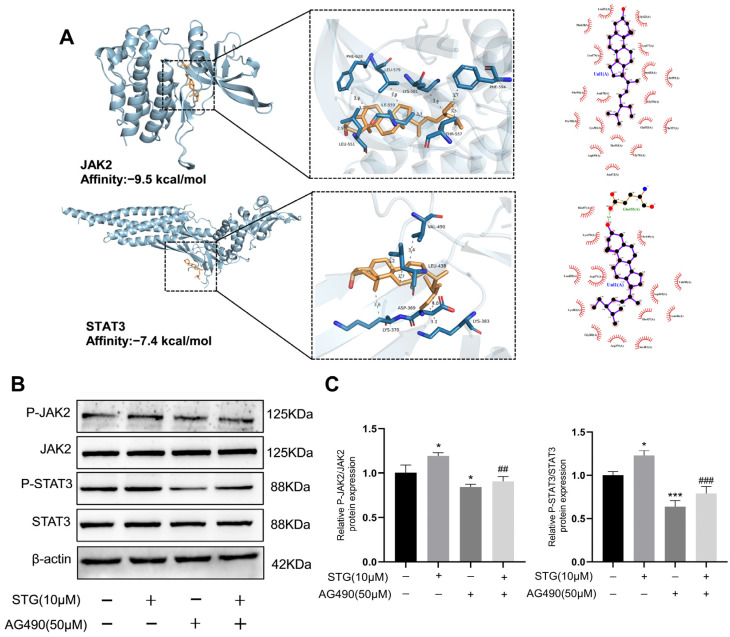
Effects of STG on the JAK2/STAT3 signaling pathway. (**A**) Molecular docking results of JAK2 and STAT3 with STG. (**B**) Expression of JAK2/STAT3 signaling pathway proteins. (**C**) Statistical analysis of JAK2/STAT3 signaling pathway protein expression. Data are shown as the mean ± SD of three independent experiments. Compared with the STG (−) AG490 (−) group, * *p* < 0.05, *** *p* < 0.001; compared with the STG (+) AG490 (−) group, ^##^ *p* < 0.01, ^###^ *p* < 0.001.

To investigate the impact of STG on the JAK2/STAT3 signaling pathway in MC3T3-E1 cells, we employed AutoDock Vina docking software to analyze the molecular interactions between STG and components of the JAK2/STAT3 pathway. Our docking analysis revealed binding energies of −9.5 kcal/mol for the STG-JAK2 interaction and −7.4 kcal/mol for the STG-STAT3 interaction (Figure 8A). Additionally, we examined the expression of relevant genes using Western blot analysis (Figure 8B,C). The results indicated that, compared to the STG (−) AG490 (−) group, the protein levels of p-JAK2 and p-STAT3 were significantly elevated in the STG (+) AG490 (−) group. These findings suggest that a STG concentration of 10 μmol/L effectively activates the JAK2/STAT3 signaling pathway. AG490 is an inhibitor of the JAK2/STAT3 signaling pathway, and it was utilized to further investigate the involvement of this pathway in the osteogenic differentiation process induced by STG. The results indicated that, compared to the STG (−) AG490 (−) group, the protein expressions of p-JAK2 and p-STAT3 in the STG (−) AG490 (+) group were significantly reduced. Furthermore, when comparing the STG (+) AG490 (−) group with the STG (+) AG490 (+) group, it was found that the reduction in the protein expression of p-JAK2 and p-STAT3 induced by STG was significantly enhanced after treatment with 50 μmol/L AG490. These findings indicate that AG490 effectively inhibits the JAK2/STAT3 signaling pathway activated by STG.

**Figure 9:**
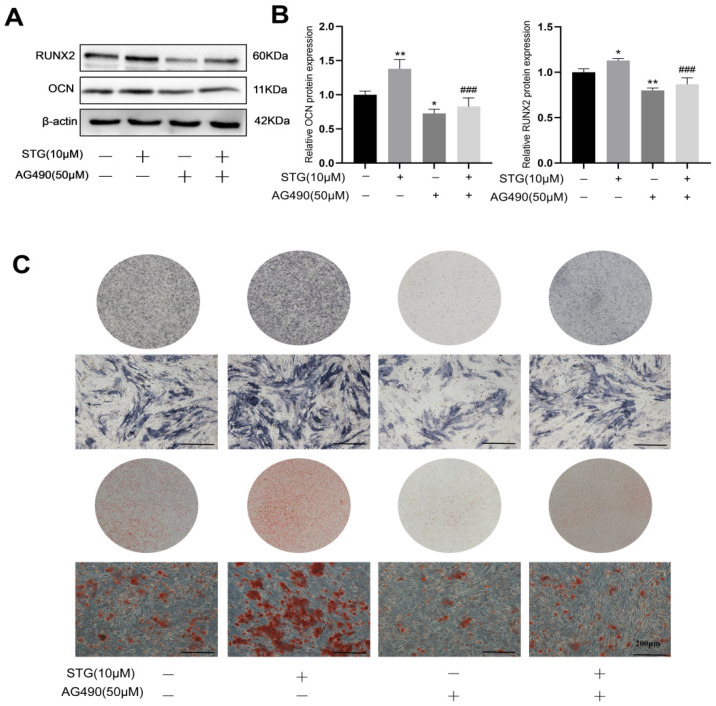
The effect of STG on osteogenic differentiation via the JAK2/STAT3 signaling pathway. (**A**) Expression of osteogenic marker proteins. (**B**) Statistical analysis of osteogenic marker protein expression. (**C**) ALP and ARS staining. Data are shown as the mean ± SD of three independent experiments. Compared with the STG (−) AG490 (−) group, * *p* < 0.05, ** *p* < 0.01; compared with the STG (+) AG490 (−) group, ^###^ *p* < 0.001.

Osteogenic assay results demonstrated that, compared with the STG (−) AG490 (−) group, the protein levels of OCN and RUNX2 were significantly elevated in the STG (+) AG490 (−) group, while the protein expression of OCN and RUNX2 was significantly reduced in the STG (−) AG490 (+) group. Compared with the STG (+) AG490 (−) group, the STG (+) AG490 (+) group also exhibited significantly reduced STG-induced protein expression of OCN and RUNX2 after treatment with 50 μmol/L AG490 (Figure 9A,B). The ALP and ARS staining results demonstrated that, compared with the STG (−) AG490 (−) group, the staining areas of ALP and ARS were significantly increased in the STG (+) AG490 (−) group, while they were significantly decreased in the STG (−) AG490 (+) group. Similarly, the staining areas were significantly decreased in the STG (+) AG490 (+) group compared with the STG (+) AG490 (−) group (Figure 9C). Thus, the JAK2/STAT3 signaling pathway may play a role in the osteogenic differentiation of MC3T3-E1 cells promoted by STG.

## 4. Discussion

This study systematically evaluated the therapeutic potential of STG for OP using an ovariectomized rat model. Micro-CT analysis revealed that STG intervention significantly reversed estrogen deficiency-induced bone microstructural deterioration, manifested as increased BMD, BV/TV, and Tb.N, alongside decreased Tb.Sp. Notably, STG not only enhanced bone mass but also improved trabecular connectivity and spatial architecture—critical for maintaining bone biomechanical strength and resilience [5]. At the model validation level, it was observed that serum E2 levels in the OVX group were significantly lower than those in the sham group, but E2 in the OVX group was not completely eliminated. This finding confirms that the sources of estrogen in the body are not limited to the ovaries; peripheral tissues such as the adrenal cortex, skin, adipose tissue, and hypothalamus also possess the ability to synthesize estrogen or convert androgens into E2 via the aromatase pathway [25,26]. Simultaneously, this indicates that the OVX model simulates a state of “relative estrogen deficiency” rather than absolute deficiency. Despite these circumstances, the significant reduction in E2 levels in the OVX group remains sufficient to establish it as a classic animal model for studying OP. STG demonstrated significant bone-protective effects even under partial estrogen deficiency, suggesting that it may act through estrogen-independent mechanisms or as a partial substitute pathway for estrogen signaling. H&E staining analysis further confirmed that STG effectively promoted trabecular bone mineralization, indicating that this compound may restore bone microstructural balance by enhancing bone formation rather than solely inhibiting bone resorption. IHC and IF detection revealed that STG restored RUNX2 and OCN expression levels in the bone marrow cavity of OVX rats. This finding aligns with the known mechanism of action of phytoestrogens: previous studies have indicated that various phytoestrogenic compounds (e.g., genistein and daidzein aglycone) promote osteoblastic differentiation by upregulating key transcription factors such as RUNX2 [5]. Furthermore, in vitro experiments demonstrated that STG significantly increased RUNX2 and OCN expression in MC3T3-E1 cells, thereby promoting osteoblast differentiation and mineralization processes.

**Figure 10:**
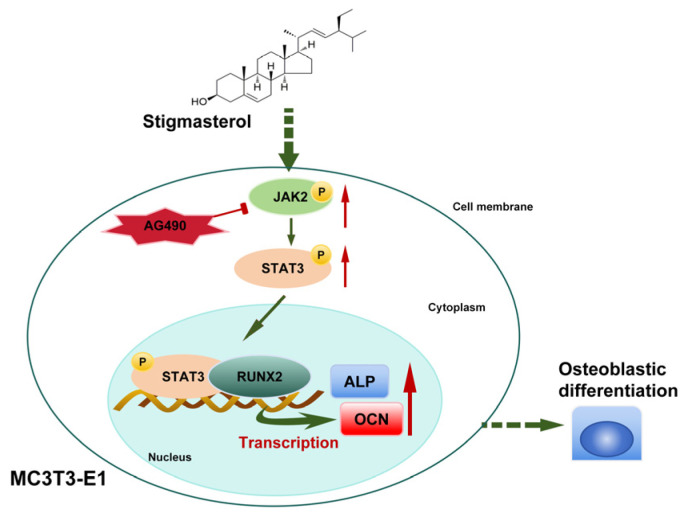
Schematic diagram of the role of STG in the JAK2/STAT3 pathway.

To systematically elucidate the molecular mechanisms underlying STG’s anti-osteoporosis effects, this study employed network pharmacology methods to predict its potential targets and signaling pathways. An analysis suggested that STG may exert its OP therapeutic effects by activating multiple key targets, including STAT3, JUN, TP53, ESR1, EGF, TNF, MYC, IL2, and JAK2. GO enrichment analysis further revealed that STG participates in multiple biological processes, including the positive regulation of kinase activity, positive regulation of MAPK cascade, enzyme-linked receptor protein signaling pathway, positive regulation of protein phosphorylation, positive regulation of cell migration, and response to oxidative stress. KEGG pathway analysis indicated that STG may exert therapeutic effects by regulating signaling pathways closely associated with bone metabolism, including PI3K-Akt, HIF-1α, MAPK, JAK-STAT, NF-κB, ErbB, and Wnt. These predictive findings collectively outline STG’s “multi-target, multi-pathway” action profile, consistent with its chemical structure as a natural plant sterol. Its multiple unsaturated double bonds and specific functional groups confer broad protein-binding capabilities. As a predictive tool, network pharmacology results require experimental validation. Therefore, this study further validated the predictions through molecular docking and in vitro cell experiments, focusing on the JAK2/STAT3 pathway. The results showed that STG exhibits stable binding conformations with both JAK2 and STAT3 proteins, with binding energies of −9.5 kcal/mol and −7.4 kcal/mol, respectively, suggesting its potential as a direct modulator of this signaling pathway. The regulatory role of the JAK2/STAT3 signaling pathway in bone metabolism exhibits cell-type specificity. Within the osteoblast lineage, activation of this pathway is typically associated with cell proliferation and early differentiation [27]. This study observed that STG significantly upregulates the phosphorylation levels of JAK2 and STAT3 in MC3T3-E1 cells, accompanied by increased expression of RUNX2 and OCN. This relationship suggests the existence of a regulatory axis: “STG → JAK2/STAT3 phosphorylation → RUNX2 transcriptional activation → OCN expression → mineralization maturation”. This inference is supported by AG490 inhibition experiments: JAK2-specific inhibition not only blocked STAT3 phosphorylation but also significantly suppressed expression of downstream osteogenic marker genes, confirming the pathway’s necessity for STG effects. This finding mechanistically aligns with that of recent studies by Wang et al. using the JAK2/STAT3 pathway inhibitor AG490 and STAT3-silencing experiments, confirming that activation of this pathway is a prerequisite for osteogenic effects [28]. However, the role of the JAK2/STAT3 signaling pathway in osteocytes is context-dependent. Notably, Xiong et al. [29] demonstrated that SDF-1/CXCR4 promotes osteogenic differentiation in BMSCs by activating JAK2/STAT3; conversely, recent studies indicate that JAK2/STAT3 activation inhibits osteogenic transdifferentiation in vascular smooth muscle cells [30]. This discrepancy may stem from cell-type-specific combinations of transcription factors or cross-talk with other signaling pathways. In this study, the findings regarding the pro-differentiating effect of STG on MC3T3-E1 pre-osteoblasts align with those of Xiong et al. [29], though the specific molecular mechanism may differ: STG may initiate the signaling cascade by directly binding to the JAK2 kinase domain rather than through indirect activation mediated by G-protein-coupled receptors (GPCRs). Furthermore, the regulatory relationship between JAK2/STAT3 and RUNX2 warrants further investigation. Although a positive correlation in their expression was observed in this study, STAT3, as a transcription factor, may regulate RUNX2 either directly (by binding to the RUNX2 promoter region) or indirectly (by modulating other transcription factors such as Osterix) [27] (Figure 10).

This study has several limitations. First, although the AG490 inhibition assay confirmed the necessity of the JAK2/STAT3 pathway, it does not rule out the possibility that STG may simultaneously act on other parallel pathways (such as Wnt/β-catenin or PI3K/Akt) [31]. Although 50 μM is the classical concentration of AG490 that effectively inhibits JAK2/STAT3 phosphorylation in various cell lines [17,32], its interaction with other kinases cannot be entirely ruled out. Moreover, prolonged high-concentration treatment may also induce nonspecific cellular stress responses. Therefore, future studies will employ more selective JAK2 inhibitors (e.g., Fedratinib) or gene knockout techniques to further validate these findings and explore precision therapeutic strategies targeting this pathway. Second, molecular docking predictions indicate that STG possesses binding affinity for both JAK2 and STAT3, yet precise binding affinity and dissociation constants (Kd) remain undetermined via biophysical methods such as surface plasmon resonance (SPR) or isothermal titration calorimetry (ITC). Finally, characteristics including STG’s in vivo bioavailability and metabolic stability will directly influence its potential for clinical translation. Future research should establish pharmacokinetic–pharmacodynamic (PK-PD) models to optimize dosing regimens.

In summary, this study integrated network pharmacology, molecular docking, and in vitro/in vivo experiments to reveal the molecular mechanism by which STG promotes osteogenic differentiation through activation of the JAK2/STAT3 signaling pathway. This discovery not only expands our understanding of plant sterols’ regulatory role in bone metabolism but also provides experimental evidence for developing novel natural product-based strategies against OP. However, translating this mechanism discovery into clinical application requires overcoming multiple challenges, including pharmacodynamic optimization, safety assessment, and formulation development.

## 5. Conclusions

Based on the findings of this study, we conclude the following: The OVX-induced OP model is reliable, and the promotion of bone formation and osteoblast differentiation by STG in OP is confirmed, which provides a foundation for further research. Through multidimensional experimental evidence, including network pharmacology prediction, molecular docking simulations, and in vitro functional validation, we demonstrate that STG enhances osteoblast differentiation and mineralization maturation by activating the “JAK2/STAT3 → RUNX2 → OCN” regulatory axis. In summary, STG holds potential as a promising osteogenic promoter and therapeutic candidate for OP.

## Figures and Tables

**Table 1:** Basic details of the top 3 subnetworks in cluster analysis.

Subnetwork	Score	Node	Target Spot
1	25.257	36	MAPK8, CD44, CTNNB1, GAPDH, STAT3, TGFB1, TNF, CREB1, MAPK14, HIF1A, MMP9, IL6, SRC, AKT1, EGFR, TLR4, CXCL10, CSF3, MAPK3, IL17A, CCL4, EGF, FN1, IL13, NFKB1, CSF2, BCL2, SIRT1, NFKBIA, IL18, CASP3, MAPK1, IL1B, PTGS2, CCL2, JUN
2	14.158	39	PARP1, IGF1R, IRS1, IL6R, ESR1, CXCL8, MTOR, PECAM1, PPARG, STAT1, VCAM1, MMP2, IL4, FOXO1, IL10, TRAF6, BCL2L1, CDKN1A, CCND1, IKBKB, TIMP1, SMAD3, TP53, RELA, PIK3R1, INS, BRCA1, GSK3B, ALB, HDAC1, ICAM1, MMP1, ERBB2, IGF1, IL2, MYC, PTEN, CSF1, MYD88
3	4.667	16	HDAC2, SMAD2, SOS1, AR, PTPN11, JAK2, HRAS, CDKN2A, SOX2, PIK3CA, NR3C1, RUNX2, PDGFRB, BDNF, CAV1, LEP

## Data Availability

The data of this study are available from the corresponding author upon reasonable request.

## Abbreviations

OP	Osteoporosis
STG	Stigmasterol
